# Radiographic outcomes of cortical screw fixation as an alternative to Kirschner wire fixation for temporary lateral column stabilization in displaced Lisfranc joint fracture-dislocations: a retrospective cohort analysis

**DOI:** 10.1186/s12891-021-04983-2

**Published:** 2022-01-17

**Authors:** Saranya A. Sethuraman, Rachel S. Silverstein, Nicket Dedhia, Adam C. Shaner, David E. Asprinio

**Affiliations:** 1 Westchester Medical Center, Department of Orthopedic Surgery, 100 Woods Road, Valhalla, NY 10595 USA; 2 Albert Einstein College of Medicine, 1300 Morris Park Avenue, Forcheimer Building, Room 251, Bronx, NY 10461 USA

**Keywords:** Lateral column, Lisfranc injury, Tarsometatarsal joint fracture-dislocation, Temporary fixation

## Abstract

**Background:**

Injuries of the tarsometatarsal joint complex ranging from purely ligamentous to multidirectionally unstable midfoot fracture-dislocations are anatomically fixed to minimize long-term sequelae including post-traumatic arthritis, pes planus deformity, and chronic pain. Lateral column disruption is commonly treated with temporary Kirschner wire (K-wire) fixation, maintaining alignment during healing and allowing resumption of physiologic motion after hardware removal. More unstable fracture patterns may require temporary cortical screw fixation to maintain adequate reduction. We evaluated the efficacy of temporary lateral column screw fixation compared to K-wire fixation for Lisfranc fracture-dislocation treatment.

**Methods:**

This retrospective cohort study reviewed 45 patients over fourteen years who underwent Lisfranc fracture-dislocation fixation at a level-one trauma center. All patients underwent medial and middle column fixation; 31 underwent lateral column fixation. Twenty six patients remained after excluding those without electronic records or follow-up. The primary outcome was radiographic lateral column healing before and after hardware removal; secondary outcomes included pain, ambulation, and return to normal shoe wear.

**Results:**

Twenty patients were male, with mean age 41 years. Thirteen patients underwent cortical screw fixation and twelve K-wire fixation. One had both implants. Twenty four patients underwent lateral column hardware removal; all had radiographic evidence of bony healing before hardware removal. Mean follow-up was 88.2 ± 114 weeks for all patients. The cortical screw cohort had significantly longer mean time to hardware removal (*p* = 0.002). The K-wire cohort had significantly more disuse osteopenia (*p* = 0.045) and postoperative pain (*p* = 0.019).

**Conclusions:**

Radiographic and clinical outcomes of unstable Lisfranc fracture-dislocation treatment support temporary lateral column screw fixation as an alternate technique.

**Level of clinical evidence:**

3 (retrospective cohort study)

## Background

The incidence of Lisfranc joint injuries is estimated at one in 55,000 people per year and 0.2% of fractures treated annually in the United States [1]. The first through third metatarsals form a trapezoidal stable arch with the second metatarsal functioning as a keystone [2]. The Lisfranc joint complex is further supported by soft tissues including a ligament complex extending from the second metatarsal to the medial cuneiform; the plantar component, or Lisfranc ligament, is strongest [2].

**Fig. 1:**
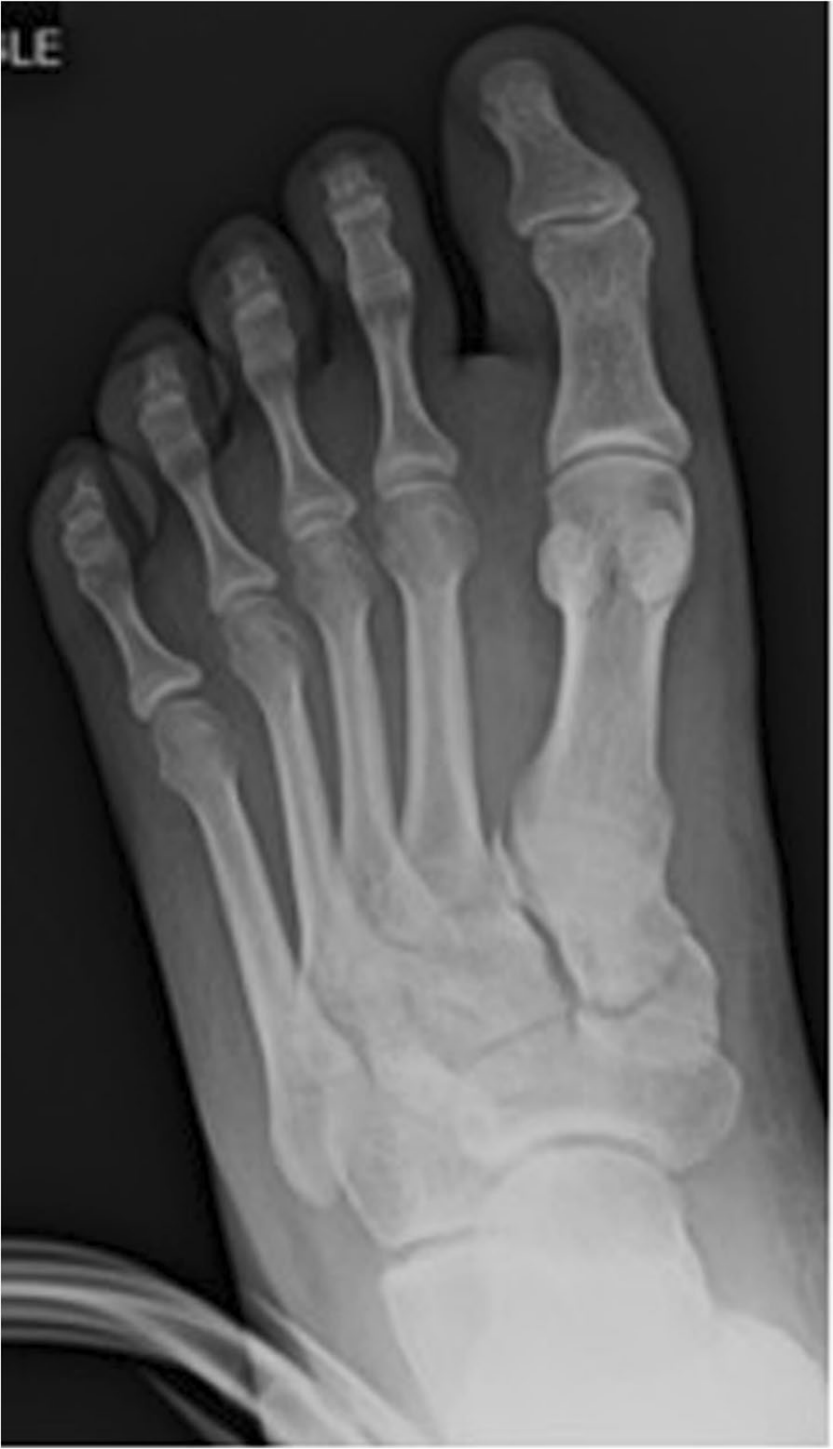
Anteroposterior radiograph of left foot demonstrating “fleck” sign pathognomonic for Lisfranc joint disruption

Lisfranc joint fracture-dislocations are typically sustained during high-energy axial loading injuries of a hyper-plantarflexed foot, or during falls from a height [3]. Avulsion of either bony attachment of the Lisfranc ligament can reveal a pathognomonic radiographic “fleck” sign (Fig. 1) [4]. The Myerson modification of the Hardcastle classification of Lisfranc injuries divides injuries into categories based on TMT joint congruity and direction of metatarsal divergence relative to the Lisfranc joint [4] and can delineate involvement of the three midfoot columns in the injury.

The lateral column has the greatest amount of sagittal plane motion and can tolerate more incongruity than the medial and middle columns [5]. The fourth and fifth TMT joints have an average 20-degree range of motion in both the dorsiflexion-plantarflexion and supination-pronation arcs [6] which allows for ambulation over uneven ground. The lateral column has historically been omitted from osseous fixation such as a TMT joint arthrodesis or open reduction and internal fixation (ORIF) to preserve motion and function of the midfoot [7–10]. If persistent instability occurs after fixation, temporary immobilization with percutaneous K-wires can help achieve a more stable construct [10]. Goals of surgical management of Lisfranc injuries include reduction to within two millimeters (mm) of articular displacement to reduce the risk of post-traumatic arthritis and chronic pes planus deformity and to restore normal gait [11].

Lateral column disruption in TMT joint injuries is commonly treated with K-wire fixation to maintain alignment during healing and allow for the return of physiologic motion after hardware removal. More unstable fracture patterns may not maintain adequate reduction with K-wire fixation; these may be treated with temporary cortical screw fixation with implant removal six to twelve weeks later. In this retrospective cohort study at a high-volume trauma center, we evaluated the efficacy of temporary lateral column cortical screw fixation as an alternative to K-wire fixation for TMT joint fracture-dislocations. The primary outcome was radiographic lateral column healing before and after hardware removal; secondary outcomes included pain, ambulation, and return to normal shoe wear. We hypothesized that temporary cortical screw fixation would yield equivalent radiographic and clinical outcomes to K-wire fixation of the lateral column.

## Methods

**Fig. 2:**
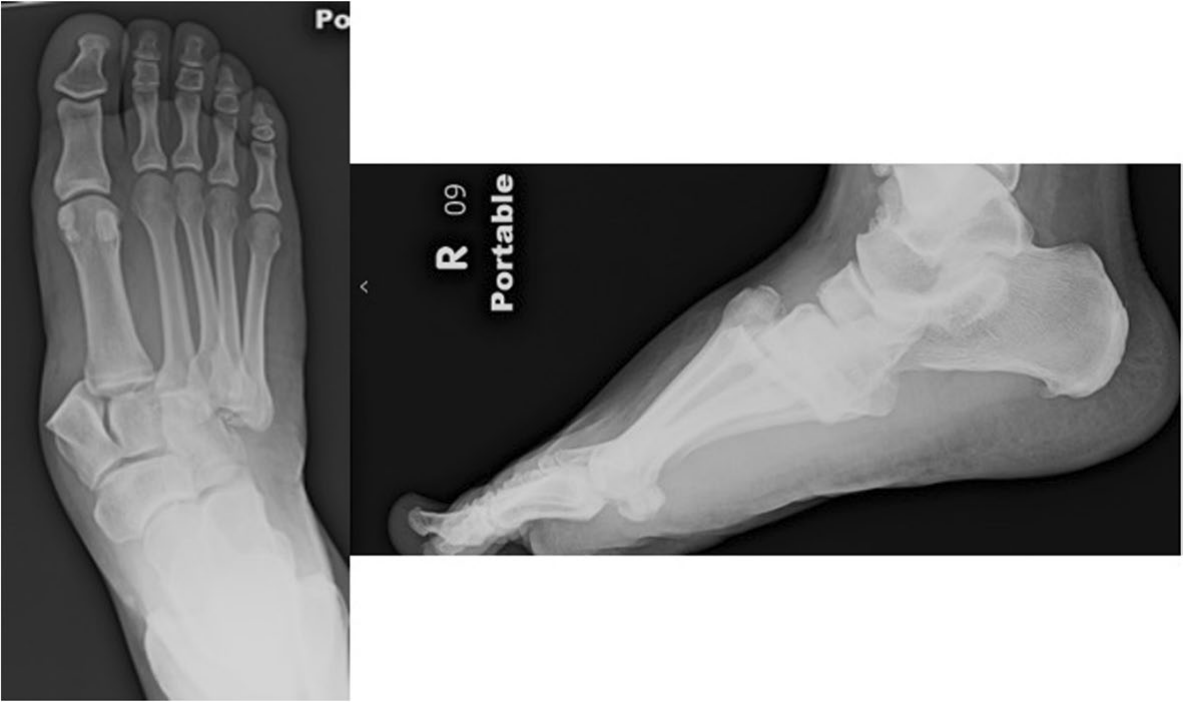
Anteroposterior and lateral injury radiographs of right foot demonstrating lateral displacement of the second metatarsal in a displaced Lisfranc joint fracture-dislocation, typical of the patients treated in this series

**Fig. 3:**
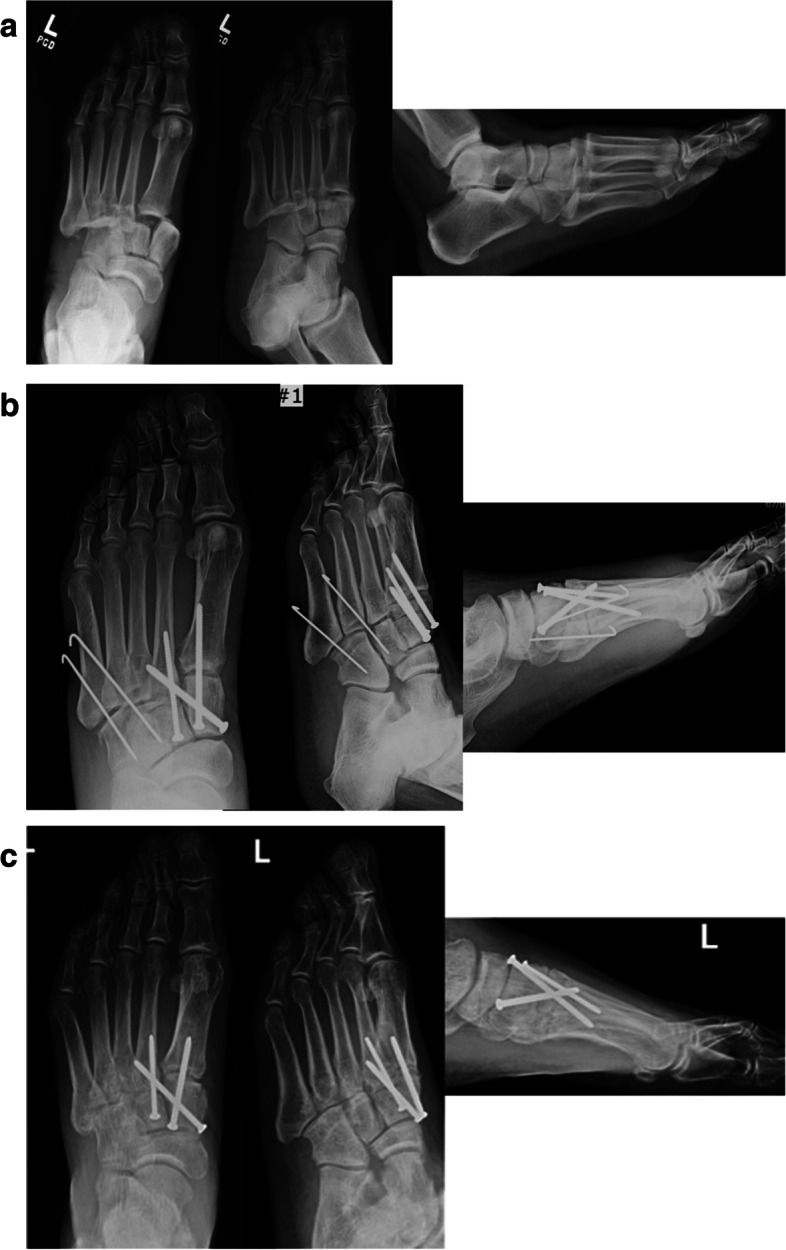
**a** Anteroposterior, oblique, and lateral injury radiographs of left foot demonstrating Myerson type A laterally incongruous TMT joint fracture-dislocation. **b** Anteroposterior, oblique, and lateral postoperative radiographs demonstrating restoration of anatomic alignment of midfoot, medial column TMT joint primary arthrodesis and lateral column K-wire fixation. **c** Anteroposterior, oblique, and lateral postoperative radiographs demonstrating removal of lateral column K-wires and mild narrowing of fourth and fifth TMT joints

**Fig. 4:**
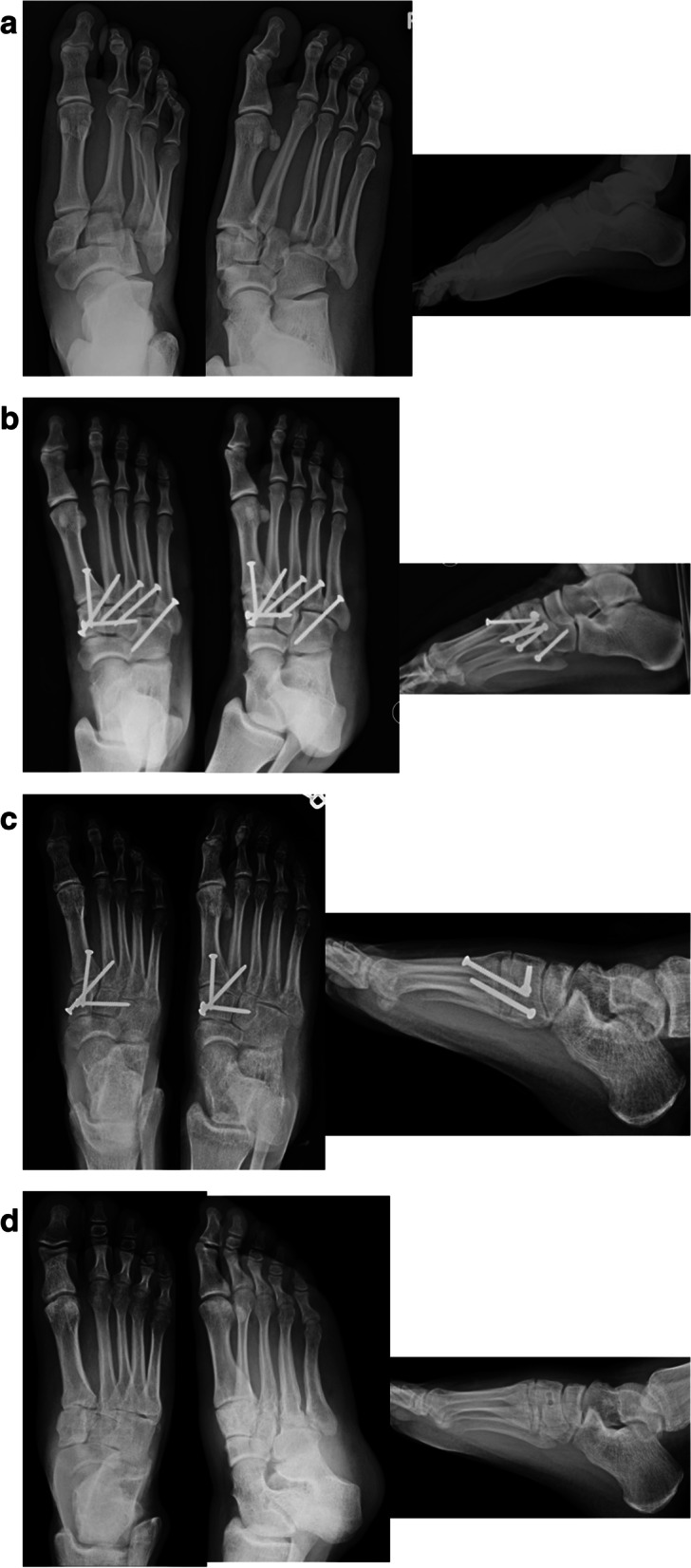
**a** Anteroposterior, oblique, and lateral injury radiographs of right foot demonstrating Myerson type C2 totally divergent TMT joint fracture-dislocation. **b** Anteroposterior, oblique, and lateral postoperative radiographs of right foot demonstrating restoration of anatomic alignment of midfoot and fixation across medial, middle, and lateral columns with cortical screws. **c** Anteroposterior, oblique, and lateral postoperative radiographs of right foot demonstrating removal of lateral column cortical screws and maintenance of anatomic alignment. **d** Anteroposterior, oblique, and lateral postoperative radiographs of right foot demonstrating removal of medial column hardware and maintenance of anatomic alignment

A retrospective cohort study was undertaken to review 45 consecutive patients from 2005 to 2018 at a level I trauma center who underwent osseous fixation of Lisfranc joint fracture-dislocations (Myerson classification types A, B1, B2, C1, and C2) by the orthopedic traumatologists. No ligamentous Lisfranc injuries were included. The New York Medical College Institutional Review Board approved this study via expedited review. Representative injury radiographs of patients treated in this cohort are included below (Fig. 2). All patients underwent medial and middle column fixation with 3.5 mm cortical screws (Synthes, West Chester, PA) and 31 also underwent lateral column fixation either with 3.5 mm cortical screws or 0.062-in. Kirschner wires (Synthes, West Chester, PA). Patients were treated according to senior author preference for lateral column fixation method. 3.5 mm or 2.7 mm plates (Synthes, West Chester, PA) were also used in medial column fixation constructs for fourteen patients. Representative pre- and post-operative radiographs of a patient fixed with K-wires as well as radiographs after hardware removal are included below (Fig. 3). Pre- and post-operative radiographs of a representative patient fixed with cortical screws in the lateral column are included for comparison (Fig. 4). After excluding those with incomplete electronic medical records and patients lost to follow up, 26 patients remained. Radiographs were assessed by two board-certified senior orthopedic traumatologists for stable hardware fixation, fracture healing, lateral column fixation method, and hardware removal. Electronic medical records were reviewed for demographic data, ambulatory status, patient-reported pain, and return to normal shoe wear. The primary outcome measure was radiographically stable lateral column healing before and after implant removal. Secondary outcomes included a pain-free foot after screw removal, ambulation without aids, and return to normal shoe wear; these were affected by other injuries sustained due to the polytraumatic nature of patients included in this study.

Chi square, Fisher’s exact, and independent samples t-tests were used to determine differences between cohorts with *p* < 0.05 considered significant. Statistical analyses were conducted with SPSS Statistics for Windows, version 25 (IBM Corp., Armonk, NY).

## Results

Thirty-one patients met inclusion criteria for this study with ages ranging from eighteen to 73 years old (mean age 39.8 ± 15.9 years). Five patients were further excluded from analysis: two patients were lost to follow up before initial postoperative evaluation, two patients underwent ipsilateral below-knee amputations within 3 days of injury, and one patient was excluded due to lack of radiographs. Twenty six patients remained eligible for analysis. Mean age for these patients was 41.0 ± 16.9 years. Six (23.1%) patients were female and fifteen (57.7%) patients had right foot injuries; no patients had bilateral Lisfranc injuries. Seven (26.9%) patients had staged hardware removal with a mean 15.7 weeks to partial hardware removal and seventeen (65.4%) patients underwent one-stage hardware removal with a mean 14.5 weeks to hardware removal. Two (7.7%) patients retained their hardware in both medial and lateral columns. Mean follow-up was 88.2 ± 114 weeks for all patients.

Twelve (46.2%) patients had lateral column disruption treated with K-wire fixation and thirteen (50.0%) were treated with screw fixation; one (3.8%) patient was treated with a combination K-wire and screw construct and was grouped in the K-wire cohort for analysis. There were no differences in age, sex, laterality, or open/closed injury status between groups (Table 1). Patients that underwent K-wire fixation were significantly more likely to have had concomitant injuries of the ipsilateral foot or ankle (including metatarsal, cuboid, navicular, talus, or distal tibia fractures) than those that underwent screw fixation (6 (50.0%) K-wire vs. 1 (7.1%) screw, *p* = 0.014).

**Table 1:** Demographic characteristics of patients that underwent lateral column fixation via cortical screw or K-wire constructs (bold font indicates significance)

	Screw (*n = 14*)	K-wire (*n = 12*)	*p*
Age (mean ± SD)	39.5 ± 17.6	42.8 ± 16.5	0.625
Sex (Female), n (%)	3 (21.4%)	3 (25.0%)	0.829
Laterality (Right), n (%)	9 (64.3%)	6 (50.0%)	0.462
Open Injury, n (%)	0 (0.0%)	1 (8.3%)	0.271
Ipsilateral Injury, n (%)	1 (7.1%)	6 (50.0%)	**0.014**

Most patients healed their fractures in both groups (13 (92.9%) screw vs. 12 (100%) K-wire, *p* = 0.345). There were no differences between groups with respect to radiographically stable fixation prior to hardware removal, need for hardware removal, staged hardware removal, development of post-traumatic arthritis, and days between surgery and weight-bearing as tolerated (*p* > 0.05) (Tables 2 and 3). Patients who underwent lateral column fixation with K-wires underwent removal of hardware in significantly fewer days postoperatively than those who underwent screw fixation (78.6 ± 29.7 days K-wire vs. 128.2 ± 37.2 days screw, *p* = 0.002). Additionally, those with K-wire fixation of the lateral column were more likely to suffer from disuse osteopenia (9 (75.0%) vs. 5 (35.7%), *p* = 0.045).

**Table 2:** Longitudinal radiographic outcomes of patients that received lateral column fixation via cortical screw or K-wire constructs (bold font indicates significance)

	Screw (*n = 14*)	K-wire (*n = 12*)	*p*
Stable Hardware, n (%)	12 (85.7%)	10 (83.3%)	0.867
Healed Fracture, n (%)	13 (92.9%)	12 (100.0%)	0.345
Hardware Removed	13 (92.9%)	11 (91.7%)	0.910
Staged Hardware Removal	5 (38.5%)	2 (18.2%)	0.276
Days to Hardware Removal (mean ± SD)	128.2 ± 37.2	78.6 ± 29.7	**0.002**

**Table 3:** Longitudinal clinical outcomes of patients that received lateral column fixation via cortical screw or K-wire constructs (bold font indicates significance)

	Screw (*n = 14*)	K-wire (*n = 12*)	*p*
Post-traumatic Arthritis, n (%)	7 (50.0%)	6 (50.0%)	1.000
Disuse Osteopenia, n (%)	5 (35.7%)	9 (75.0%)	**0.045**
Days to weight-bearing as tolerated (mean ± SD)	131.8 ± 40.1	133 ± 82.2	0.961
Unaided Mobility, n (%)	13 (92.9%)	8 (80.0%)	0.348
Return to Normal Shoe Wear, n (%)	13 (92.9%)	12 (100.0%)	0.345
Post-operative Pain, n (%)	0 (0.0%)	4 (33.3%)	**0.019**

Functional outcomes assessed at latest follow-up by the senior orthopaedic traumatologists, including return to unaided mobility and return to normal shoe wear, did not differ significantly between groups (both *p* > 0.05). One patient in the cortical screw cohort required a one-inch heel lift post-operatively and two patients in the K-wire cohort required a cane for ambulatory assistance due to a peroneal palsy and ipsilateral cuboid and talus fractures, respectively. Despite requiring an ambulatory aid and specialist shoe wear, one patient reported no pain. Patients that underwent lateral column K-wire fixation were more likely to complain of post-operative pain than those that underwent screw fixation (4 (33.3%) vs 0 (0.0%), *p* = 0.019), perhaps related to the increased rates of ipsilateral injury noted in that group.

## Discussion

Both fixation methods used in this case series achieved the primary outcome of stable radiographic healing of the lateral column of the midfoot. Both fixation methods also achieved a return to normal shoe wear and a return to mobility without assistive devices. Notably, those fixed with K-wires were able to undergo lateral column hardware removal an average of 7.1 weeks sooner than those fixed with cortical screws despite similar demographic characteristics and an increased frequency of ipsilateral injuries, potentially due to K-wire removal during office consultation rather than during a subsequently scheduled return to the operative suite. Lateral column screw fixation was associated with less postoperative pain and less disuse osteopenia although there was no significant difference in return to full weight-bearing between groups. Additional potential benefits of lateral column screw fixation not specifically evaluated in this study include avoiding exposed hardware and reducing urgency of hardware removal, reducing the risk of pin tract infection, and allowing accelerated patient hygiene. The cortical screw technique requires no additional surgical skills. Screw fixation avoids the need for pin care but does necessitate a second surgical procedure while K-wires may be removed in the office setting.

Other techniques using K-wires in the lateral column were attempting to retain some of the motion present in those joints [10]. In more severely displaced fracture-dislocations, screws may be a better choice to maintain lateral column reduction. A primary driving force of the development of post-traumatic arthritis is inadequate reduction [11–13]; therefore, the best surgical technique is that which affords stable anatomic reduction until fracture healing.

Recent studies have supported primary arthrodesis of the Lisfranc joint as a treatment method for acute ligamentous Lisfranc injuries, with a systematic review in 2015 suggesting that primary arthrodesis was equivalent to ORIF [14]. Ly and Coetzee reported a prospective randomized study comparing primary arthrodesis with ORIF of primarily ligamentous Lisfranc injuries involving the medial and middle columns [15]. With 2 years of follow up, they found arthrodesis patients to have 92% of their pre-injury function, while internal fixation patients had 65% of their pre-injury function [15]. A 2019 Australian case series of 39 patients with osseous Lisfranc fracture-dislocations suggests improved outcomes with primary arthrodesis of the medial and middle columns when compared to ORIF in patients with totally incongruous TMT joint injuries (Myerson types A and C2) [16]. Temporary lateral column fixation was performed with K-wires in 80.9% of the ORIF group and 55.6% of the arthrodesis group [16]. However, only 62% of the ORIF group had anatomic reduction at index surgery and 53% of those patients lost reduction at follow up [16], suggesting more study is needed to delineate outcomes between arthrodesis and ORIF in severe Lisfranc joint fracture-dislocations. Regardless of the fixation method chosen for the medial and middle columns of the midfoot, lateral column stabilization appears to be necessary in severe Lisfranc fracture-dislocations, and this case series demonstrates that cortical screws and K-wires are equivalent techniques for achieving that goal.

This case series includes limitations, including the retrospective nature of data collection; randomizing and prospectively following patients may provide additional insight differentiating the two techniques. Additionally, use of other metrics including those encompassing a wider variety of activities of daily living would allow for additional clinical outcomes analysis. A prospective study collecting patient-reported outcomes measures using K-wire and cortical screw fixation techniques would build on this radiographic study to provide further clinically significant data, yet may be difficult to implement due to the relative rarity of this complex injury pattern. Despite these constraints, this study demonstrates that lateral column cortical screw fixation is a viable alternative with equivalent radiographic and possibly improved functional outcomes to K-wire fixation for temporary immobilization of the fourth and fifth TMT joints in displaced Lisfranc fracture-dislocations.

## Conclusions

This report demonstrates that temporary lateral column fixation in unstable Lisfranc joint injuries using cortical screws provides equivalent radiographic outcomes to the current standard of care of K-wire fixation, and does not delay return to weight-bearing, unaided mobility, and normal shoe wear.

## Data Availability

The datasets analyzed are available from the corresponding author upon request.

## Abbreviations

K-wire: Kirschner wire
TMT: Tarsometatarsal
ORIF: Open reduction internal fixation

## References

[1] Desmond EA, Chou LB (2006). Current concepts review: Lisfranc injuries. Foot Ankle Int.

[2] Moracia-Ochagavía I, Rodríguez-Merchán EC (2019). Lisfranc fracture-dislocations: current management. EFORT Open Rev.

[3] Watson TS, Shurnas PS, Denker J (2010). Treatment of Lisfranc joint injury: current concepts. J Am Acad Orthop Surg.

[4] Myerson MS, Fisher RT, Burgess AR, Kenzora JE (1986). Fracture dislocations of the tarsometatarsal joints: end results correlated with pathology and treatment. Foot Ankle.

[5] Scolaro J, Ahn J, Mehta S (2011). Lisfranc fracture dislocations. Clin Orthop Relat Res.

[6] Ouzounian TJ, Shereff MJ (1989). In vitro determination of Midfoot motion. Foot Ankle.

[7] Quénu E, Küss G (1909). Etude Sur les luxations du métatarse (luxations métatarso-tarsiennes). Du diastasis entre le 1er et le 2e métatarsien. Rev Chir.

[8] Cassebaum WH (1963). Lisfranc fracture-dislocations. Clin Orthop Relat Res.

[9] Arntz CT, Hansen ST (1987). Dislocations and fracture dislocations of the tarsometatarsal joints. Orthop Clin North Am.

[10] Stavlas P, Roberts CS, Xypnitos FN, Giannoudis PV (2010). The role of reduction and internal fixation of Lisfranc fracture–dislocations: a systematic review of the literature. Int Orthop.

[11] Lau S, Guest C, Hall M, Tacey M, Joseph S, Oppy A (2017). Functional outcomes post Lisfranc injury-Transarticular screws, dorsal bridge plating or combination treatment?. J Orthop Trauma.

[12] Calder JDF, Whitehouse SL, Saxby TS (2004). Results of isolated Lisfranc injuries and the effect of compensation claims. J Bone Joint Surg (Br).

[13] Arntz CT, Veith RG, Hansen ST (1988). Fractures and fracture-dislocations of the tarsometatarsal joint. J Bone Joint Surg Am.

[14] Smith N, Stone C, Furey A (2015). Does open reduction and internal fixation versus primary arthrodesis improve patient outcomes for Lisfranc trauma? A systematic review and meta-analysis. Clin Orthop Relat Res.

[15] Ly TV, Coetzee JC (2006). Treatment of primarily ligamentous Lisfranc joint injuries: primary arthrodesis compared with open reduction and internal fixation. A prospective, randomized study. J Bone Joint Surg Am.

[16] Kirzner N, Teoh W, Toemoe S, Maher T, Mannambeth R, Hughes A (2019). Primary arthrodesis versus open reduction internal fixation for complete Lisfranc fracture dislocations: a retrospective study comparing functional and radiological outcomes. ANZ J Surg.

[17] Sethuraman SA, Silverstein RS, Dedhia N, Shaner AC, Asprinio DE. (2021). Cortical screw fixation as an alternative to Kirschner wire fixation for temporary lateral column stabilization in displaced Lisfranc joint fracture-dislocations [conference presentation abstract]. Thirty-seventh Annual Meeting of the Orthopaedic Trauma Association, Fort Worth, Texas, United States. https://ota.org/sites/files/2021-10/1015-PRF8-FNL%20Screen-AM21%20Fnl%20Program.pdf.

